# Conformational flexibility related to enzyme activity: evidence for a dynamic active-site gatekeeper function of Tyr^215^ in *Aerococcus viridans* lactate oxidase

**DOI:** 10.1038/srep27892

**Published:** 2016-06-15

**Authors:** Thomas Stoisser, Michael Brunsteiner, David K. Wilson, Bernd Nidetzky

**Affiliations:** 1 Research Center Pharmaceutical Engineering, Inffeldgasse 13, A-8010 Graz, Austria; 2 Graz University of Technology, Institute of Biotechnology and Biochemical Engineering, NAWI Graz, Petersgasse 12, A-8010 Graz, Austria; 3 Department of Molecular and Cellular Biology, University of California, Davis, CA 95616, USA

## Abstract

L-Lactate oxidase (LOX) belongs to a large family of flavoenzymes that catalyze oxidation of α-hydroxy acids. How in these enzymes the protein structure controls reactivity presents an important but elusive problem. LOX contains a prominent tyrosine in the substrate binding pocket (Tyr^215^ in *Aerococcus viridans* LOX) that is partially responsible for securing a flexible loop which sequesters the active site. To characterize the role of Tyr^215^, effects of substitutions of the tyrosine (Y215F, Y215H) were analyzed kinetically, crystallographically and by molecular dynamics simulations. Enzyme variants showed slowed flavin reduction and oxidation by up to 33-fold. Pyruvate release was also decelerated and in Y215F, it was the slowest step overall. A 2.6-Å crystal structure of Y215F in complex with pyruvate shows the hydrogen bond between the phenolic hydroxyl and the keto oxygen in pyruvate is replaced with a potentially stronger hydrophobic interaction between the phenylalanine and the methyl group of pyruvate. Residues 200 through 215 or 216 appear to be disordered in two of the eight monomers in the asymmetric unit suggesting that they function as a lid controlling substrate entry and product exit from the active site. Substitutions of Tyr^215^ can thus lead to a kinetic bottleneck in product release.

A large and widely distributed family of flavoenzymes is involved in the biological oxidation of α-hydroxy acid substrates in various organisms and cell types^1^,^2^. Activity in theses enzymes depends on their flavin mononucleotide (FMN) cofactor. Common characteristics of their structure are the (β/α)_8_ barrel fold and arrangement of protein subunits into a functional homotetramer^2^. The FMN is bound at the C-terminal end of the internal β barrel. The α-hydroxy acid-oxidizing flavoenzymes have attracted significant interest to elucidate basic principles of FMN-dependent enzymatic catalysis^1^,^2^,^3^ [for general reviews, see ref. 4,5]. Moreover, the enzymes have been considered for different applications^6^, particularly in the biocatalytic synthesis of chemicals^7^,^8^ as well as in biosensing^9^,^10^.

The *Aerococcus viridans* L-lactate oxidase (avLOX) is a biotechnologically important member of this flavoenzyme family and it catalyzes oxidation of L-lactate and O_2_ into pyruvate and H_2_O_2_^1^,^2^,^3^,^5^,^11^. A major clinical use of avLOX is in the determination of L-lactate indirectly via the H_2_O_2_ formed in the reaction^9^,^10^. The overall conversion of L-lactate occurs in two half-reactions, typical of enzymes from this family^2^,^5^ and as shown in Fig. 1, where the FMN cofactor is reduced by an α-hydroxy acid substrate and then re-oxidized by O_2_^1^,^3^,^11^,^12^,^13^. Mechanistically, His^265^ is thought to abstract the proton from the α-OH of L-lactate to facilitate hydride transfer from substrate to N5 of FMN (Fig. 2)^14^,^15^,^16^,^17^,^18^,^19^,^20^,^21^,^22^,^23^. A crystal structure of oxidized avLOX bound with pyruvate^24^ has revealed an active-site group arrangement similar to that seen in other known members of the α-hydroxy-acid oxidase family of flavoenzymes^25^,^26^ where a Tyr/Tyr/Arg triad of conserved residues (Tyr^40^, Tyr^146^ and Arg^268^ in avLOX) appears to be critical for positioning the L-lactate substrate for catalysis. Figure 2 shows that Tyr^40^ and Arg^268^ create a binding pocket for the pyruvate carboxylate group whereas Tyr^146^ interacts with the C2 keto group. Targeted mutagenesis of homologous residues in related flavoenzymes (e.g. L-lactate monooxygenase^27^,^28^, glycolate oxidase^29^,^30^, L-mandelate dehydrogenase^31^,^32^, flavocytochrome *b*_5_^33^,^34^) suggested catalytic function of the active-site triad in the stabilization of the first half-reaction’s transition state where enzyme is reduced by α-hydroxy acid substrate.

A special and interesting feature of the catalytic center of avLOX is that it contains a third tyrosine (Tyr^215^), which forms a weak hydrogen bond with the pyruvate O2 in the wild-type structure (Fig. 2)^24^. Moreover, it is also partially responsible for closing loop 4, the sequence of residues consisting of the residue range 173–222 which connect β4 and α4 in the core (β/α)_8_ barrel, over the active site via its interaction with the lactate and side chains on the core of the protein such as Arg^181^. Movement of all or part of this flexible loop is necessary for substrate binding and product release^12^,^14^,^24^,^35^,^36^,^37^. It was therefore particularly interesting to determine what specific role Tyr^215^ might play in the catalytic reaction of avLOX and especially, in the proposed loop conformational change involved in product dissociation. Clearly, the loop opening would have to ensure that the catalytic steps of flavin reduction and oxidation are properly timed with the physical step of α-keto-acid product release. Despite high similarity in their active-site architectures, other α-hydroxy-acid oxidases catalyze distinctly different chemical transformations (e.g. oxidase and dehydrogenase types of oxidation; oxidative decarboxylation)^1^,^2^,^3^,^12^,^13^,^27^,^28^. Determinants of reaction selectivity are therefore likely to be the non-conserved residues in the active site such as Tyr^215^. How the protein structure controls the enzymatic reactivity is an important but elusive problem of the α-hydrody acid-oxidizing flavoenzyme family as a whole. Deepened understanding of how residue variations affect the enzymatic reaction pathway of α-hydroxy acid oxidation is not only a fundamental goal in the study of this group of flavoenzymes but it could also contribute to the development of novel oxidation biocatalysts with improved or altered reactivities for biotechnological use^6^.

## Results

The two variant lactate oxidases were purified by the same protocol as described previously for wild-type avLOX^12^. The purified Y215F and Y215H mutants showed a similar absorbance spectrum to the wild-type enzyme in the visible and ultraviolet wavelength regions (Fig. 3).

### Steady-state kinetic analysis

Kinetic parameters for L-lactate oxidation by wild-type and variant enzymes were determined under air-saturated conditions ([O_2_] = 250 μM) at pH 6.5 and 20 °C. In terms of their apparent *k*_cat_, Y215F (*k*_cat_ = 22 ± 2 s^−1^) and Y215H (*k*_cat_ = 5.0 ± 0.1 s^−1^) were 4-fold and 18-fold less active than the wild-type enzyme (*k*_cat_ = 88 ± 2 s^−1^), respectively. The *K*_m_ for L-lactate was decreased 4-fold in Y215F (*K*_m_ = 0.13 ± 0.01 mM) and unchanged in Y215H (*K*_m_ = 0.52 ± 0.12 mM) compared to the wild-type *K*_m_ ( = 0.50 ± 0.10 mM). Besides *k*_cat_, the catalytic efficiency (*k*_cat_/*K*_m_) is the key steady-state parameter. *k*_cat_/*K*_m_ was similar to wild-type (176 ± 31.2 mM^−1^s^−1^) in the Y215F variant (169 ± 2.36 mM^−1^s^−1^) whereas it was decreased about 18-fold in Y215H (9.61 ± 2.02 mM^−1^s^−1^). Kinetic data to be presented later can be used to calculate the *K*_m_ for O_2_. Values of 51 μM, 24 μM and 62 μM were calculated for wild-type enzyme, Y215F and Y215H, respectively. Therefore, these results suggest that kinetic parameters were determined at a somewhat saturating concentration of O_2_ (≥4 × *K*_m_). They also suggest that effect of the site-directed substitutions on the *K*_m_ for O_2_ was relatively small.

### Kinetics of the reductive half-reaction with L-lactate

When Y215F and Y215H were mixed with L-lactate under anaerobic conditions in the stopped-flow instrument, the absorbance spectrum of the enzymes changed as shown in Fig. 3, indicating reduction of the FMN cofactor over time. The wild-type enzyme was analyzed in previous studies^12^,^37^ and it shows similar behavior (see Figure S1, Supporting Information). Decrease in absorbance at 455 nm was used to monitor the enzyme reduction (Fig. 3B,C). At all L-lactate concentrations used (0.1–10 mM), loss of the 455 nm absorbance and hence the enzyme reduction appeared to be complete in both lactate oxidase variants. Reaction of the Y215F variant with L-lactate was additionally characterized by a fast intermittent appearance of absorbance at ≥500 nm (maximum around 530 nm) that decreased again afterwards only to become lost completely at longer times (Fig. 3A). Time-resolved absorbance spectra of Y215H did not show absorbance changes at ≥530 nm during enzyme reduction (Fig. 3C). In accordance with previous studies of avLOX^1^,^12^,^13^, absorbance at 530 nm is assigned to the reduced enzyme-pyruvate complex of Y215F whose formation and decay was clearly resolved with time, as shown in the inset of Fig. 3B. Enzyme reduction rate constants (*k*_obs_) were obtained from exponential or bi-exponential fits of the time traces at 455 nm. Figure 4 shows the dependencies of *k*_obs_ on the L-lactate concentration. Kinetic parameters determined from the stopped-flow data are summarized in Table 1.

Stopped-flow traces of the Y215F variant at 455 nm were biphasic. A first phase of rapid absorbance decay was followed by a phase in which the absorbance disappeared more slowly, as shown in Fig. 3B. The *k*_obs_ associated with the fast and slow reduction phase differed by ≥3-fold at L-lactate concentrations of 0.5 mM and greater, thus allowing for an independent determination of each rate parameter. As shown in Fig. 4A, the first phase was L-lactate concentration-dependent whereas the second phase appeared to be independent of L-lactate concentration. The kinetic measurements yielded the reduction rate of the Y215F variant (124 s^−1^) to be just slightly (2.2-fold) decreased compared to that obtained for the wild-type enzyme (270 s^−1^) as shown in Table 1. From the hyperbolic dependence of *k*_obs_ on L-lactate concentration (Fig. 4A) the apparent dissociation constant *K*_d_ of L-lactate binding to Y215F was determined to be 0.28 mM versus 1.0 mM determined for the wild type (Table 1). The second phase of the stopped-flow traces of the Y215F variant yielded the rate constant of pyruvate release from the reduced enzyme (20 s^−1^), which was independent of the L-lactate concentration. By combining kinetic simulation and fitting as shown in Fig. 3B (inset), the time trace at 530 nm was used to obtain a reduction rate constant of 150 s^−1^ and a rate constant of pyruvate release of 17 s^−1^, both in good agreement with the results in Table 1.

As with wild-type enzyme, time traces of the Y215H variant showed disappearance of absorbance at 455 nm in only a single kinetic phase. The associated *k*_obs_ showed hyperbolic dependence on the L-lactate concentration (Fig. 4B). The enzyme reduction rate (8.2 s^−1^) obtained from the data was decreased 33-fold as compared to wild-type enzyme (Table 1).

### Kinetics of the oxidative half-reaction with O_2_

The lactate oxidase variants were reduced anaerobically by L-lactate and their reoxidation was measured at different O_2_ concentrations in the stopped-flow instrument. Time-resolved analysis of absorbance spectra revealed that enzyme reoxidation progressed to completion for each lactate oxidase variant under all conditions used (Figure S2-A). Increase in absorbance at 455 nm due to FMN oxidation occurred in a single phase from which the *k*_obs_ was determined (Figure S2-A, inset). As with wild-type avLOX, the *k*_obs_ for each variant showed a linear dependence on the O_2_ concentration used (Figure S2-B,C). The second order rate constant *k*_ox_ was obtained as the slope of a straight-line fit to the data and the results are summarized in Table 1. The *k*_ox_ is a measure of the O_2_ reactivity of the reduced oxidase. It was decreased 2.6- and 18-fold, compared to *k*_ox_ of wild-type enzyme, in the Y215F and the Y215H variant, respectively.

### pH effects on the reductive and the oxidative half-reactions

Stopped-flow experiments were performed to determine rates of FMN reduction and oxidation at below (pH 5.5) and above (pH 9.5) the pH range where wild-type and mutated lactate oxidases exhibit their optimum activity (pH 6.5–7.5). Table 1 summarizes rate constants obtained from fits of the stopped-flow traces. Unlike enzyme reduction by L-lactate in wild-type avLOX at pH 6.5 and pH 5.5 which proceeded in a single kinetic phase, reaction at pH 9.5 appeared to be biphasic where a fast initial reduction phase was followed by a much slower but still significant second phase of enzyme conversion (Fig. 5A). The second phase contributed about 10% of the total absorbance change at 455 nm, which was just sufficient for this phase to be analyzed. The rate constant of the fast phase was dependent on the L-lactate concentration, as shown in Fig. 5B. The rate constant of the slow phase was independent of the L-lactate concentration (Fig. 5B).

For the Y215F variant, time traces of enzyme reduction were biphasic at each pH. *k*_obs_ of the fast reaction phase decreased at pH 5.5 and 9.5 as compared to pH 6.5 whereas the *k*_obs_ of the slow reduction phase was not pH-dependent. The *k*_obs_ of the single-phase reduction of Y215H mutant was also independent of pH. It was also determined that the *k*_ox_ of the wild-type enzyme was decreased 1.6- and 3.0-fold at pH 5.5 and pH 9.5 as compared to pH 6.5, respectively. The *k*_ox_ of the enzyme variants was only measured at pH 6.5.

### Crystal structure of the Y215F variant of avLOX

Crystals of the Y215F mutant grew in the *P*2_1_ spacegroup and had two tetramers within the asymmetric unit (Table 2). Overall B-factors ranged from 21.3 to 28.2 Å^2^ for the various subunits. Measurements were made on monomer B, which had the lowest B-factor unless otherwise noted. The refined 2.6 Å structure shows the expected central (β/α)_8_ barrel motif and that the overall r.m.s.d. between C_α_ pairs ranged from 0.2–0.4 Å for each subunit when compared to subunit B of the wild-type enzyme (PDB code: **2E77**)^23^,^24^. Also present in each subunit is the FMN cofactor and a pyruvate which both bind in a similar manner to the wild-type enzyme (Fig. 6, Figure S3). Several Ramachandran outliers involving Gln269 and Ser297 were noted which have strong corresponding electron density. Similar conformations have been seen for these residues in other high resolution avLOX structures^12^,^14^,^23^,^24^,^35^,^36^,^37^.

Since the pyruvate is almost completely sequestered from solvent, there is an implied conformational change necessary for binding of substrate and release of product. Residues 200–215 in subunit D and 200–216 in subunit H, which reside in loop 4 had no corresponding interpretable density. These residues form a short helical structure, which could function as an active-site lid, and residues near this range have been observed to be disordered in other structures suggesting an inherent mobility. The effects of this mobility can be seen when structures within the asymmetric unit are overlaid (Fig. 6). In the six subunits with observable loop residues, the pyruvate is bound in a manner very similar to the wild-type enzyme except for a difference in the interaction with the amino acid at position 215. The wild-type enzyme binds the pyruvate with a hydrogen bond between the phenolic hydroxyl and the O_2_ on the pyruvate. Although the distance is short (2.4 Å), the hydrogen bond is also very poor in geometry resulting in an interaction, which is not likely to be very strong. Furthermore, Tyr^215^ acts as a hydrogen bond acceptor with Arg^181^ on the opposing side of the binding side, which is likely to aid in fastening the lid. The interaction in the enzyme variant positions the nearest Phe^215^ ring atom within ~3.5 Å of the pyruvate methyl group to replace the hydrogen bond with a hydrophobic interaction. The hydrogen bond with Arg^181^ is also lost.

In the two subunits without observable density for the loop, the pyruvate maintains some of the interactions between its carboxylate and Arg^268^ and Arg^181^ but shifts in the pyruvate and both of these side chains result in weaker interactions. Hydrogen bonding interactions between the pyruvate carbonyl oxygen and His^265^ are also broken (Fig. 6). This allows the pyruvate methyl group to rotate away from the plane of the FMN isoalloxazine ring, a potential first step in dissociation. A further comparison of the structures of the loop-closed and loop-open subunits reveals >1 Å shifts of the side chains of other residues in or near the active site including Tyr^124^, Tyr^191^ and Met^195^.

### Molecular Dynamics Simulations

100-ns MD simulations were performed on enzyme-pyruvate complexes of wild-type avLOX and the Y215F variant at pH 7.0. For wild-type enzyme, the simulation was also done at a high pH of 9.5, setting Tyr^215^ to a deprotonated state. Analysis of the r.m.s. deviation of trajectories calculated from simulation data showed that each enzyme converged readily into an apparently stable structure (≤2 Å r.m.s.d.) that was highly similar to the experimental reference structure (Fig. 7A). Slight changes in the wild-type structure, particularly in the region of the active-site lid, were noted to occur as a result of the increase in pH from 7.0 to 9.5. Figure 7B shows a frequency distribution of distances between the guanidino Cζ of Arg^181^ and the phenyl Cζ of Tyr^215^/Phe^215^ for the whole 100 ns simulation trajectory. In wild-type enzyme at each pH, a narrow and well-defined frequency distribution was obtained, indicating that the two C atoms adopted a fairly stable position relative to each other. The average distance was about 1.2 Å shorter at pH 9.5 than at pH 6.5. In the Y215F variant, by contrast, the interatomic distance varied in a broad range and was mostly larger than it was in the wild-type enzyme irrespective of whether a pH of 6.5 or 9.5 was used. Analysis of simulated trajectories for the individual residues showed that the enhanced fluctuation in distance in the Y215F variant was mainly due to a conformational flexibility of Phe^215^ that was strongly augmented as compared to Tyr^215^.

Based on results of the MD simulation a theoretical titration curve for Tyr^215^ was calculated (Fig. 7C). A side chain p*K*_a_ of around 9.0–9.5 was estimated from the data. Therefore, this suggests that Tyr^215^ is (partly) ionized at a reaction pH of 9.5, thus supporting the MD simulation done with the tyrosine side chain deprotonated.

Besides the tightening of the hydrogen bond between Arg^181^ and Tyr^215^, the enzyme simulated with a deprotonated Tyr^215^ at high-pH conditions showed slight changes in the positions of the two interacting residues. Furthermore, it showed positional shifts of two helices (residues 182–187, bottom left; residues 211–217, bottom center) as well as conformational changes of the loop containing Arg^181^ (Fig. 7D). The bound pyruvate was also positioned differently at pH 9.5 compared to pH 7.0, featuring position changes in both the keto group and the carboxy group. The protein conformation change at high pH in the modeled structure resulted in an increase in the space around pyruvate, which was occupied by 3–4 water molecules (not shown in Fig. 7D for clarity). The MD simulations suggest that the observed slowing down of pyruvate release at pH 9.5 (see the Discussion) might be a consequence of conformational rearrangements promoted at high pH. Strengthened interaction between Arg^181^ and Tyr^215^ appears to be key element of the overall conformational change.

## Discussion

Results are presented here that support a role of Tyr^215^ in linking protein conformational flexibility to enzymatic activity of avLOX. The tyrosine is suggested to participate in control of opening/closing motions of the active-site lid in avLOX. This is important not only for effective turnover by avLOX but also for balanced binding of L-lactate substrate and pyruvate product. Evidence of the relevance of protein dynamics at the millisecond time scale to the catalytic pathway appears to be of importance in a family-wide context of α-hydroxy acid-oxidizing flavoenzymes. This is because the structural element of the active-site lid is conserved, even though Tyr^215^ and the specific interactions of this residue are present only in avLOX. Moreover, the relation between conformational dynamics and enzyme catalysis is of currently high interest in enzyme research in general^38^,^39^,^40^,^41^.

It remains difficult to give a clear-cut definition of the residues which function as a moveable loop based on disorder observed within structures. Prior studies of avLOX have shown that subunits are missing all or parts of the following segments of residues: 199–212, 200–209, 198–217^36^, 196–213, 200–211^24^, 176–217, 177–214^14^ and 200–215 and 200–216 in the current lactate oxidase variant structure. Although it is possible that this variability may be due to the difference in packing forces arising from the different crystal forms, the potential exists that there are no unique hinge residues and that there could be multiple modes of product exit from the active site. The range of amino acids from approximately 200 through 215 appears to be commonly disordered throughout most of these models. The range 200–210 is composed of a loop of residues of which only three engage in direct polar interaction with the core of the protein. Following this loop is a short helix ending with Tyr^215^, which forms a hydrogen bond with Arg^181^, one of the few polar interactions between the potential “lid” and a core residue. This region of the helix is closest to the pyruvate binding site and it is therefore likely that all or part of it will move during product exit from the active site. The hydrogen bond between Tyr^215^ and Arg^181^ would therefore be among the interactions that must be broken in a conformational change involving this helix.

Substitution by phenylalanine at this position probably leads to a slower dissociation of product (see below for discussion of kinetics) presumably due to a tighter lid interaction. Although the weak hydrogen bond to the pyruvate O2 and another hydrogen bond to Arg^181^ are lost, a hydrophobic interaction with the pyruvate methyl group is gained as a result of the pyruvate re-orienting relative to the wild-type enzyme (Fig. 2). Moreover, partitioning of the phenylalanine sidechain into solvent is much more unfavorable than a tyrosine when the lid is in the open conformation. Similar effects of partitioning lid residues on product off rates have been noted before in avLOX variants^37^. The change to a histidine at this position also results in a reduction of product off rate. We speculate that side chain rearrangements would allow establishment of hydrogen bonding between the histidine side chain and serines at positions 175 and/or 178. Either of these potential interactions would aid in securing the loop over the active site.

**Figure 1:**
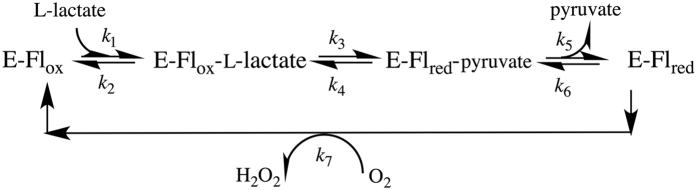
Proposed reaction pathways of L-lactate oxidase where E-Fl_ox_ and E-Fl_red_ are the enzyme forms containing oxidized and reduced FMN, respectively.

**Figure 2:**
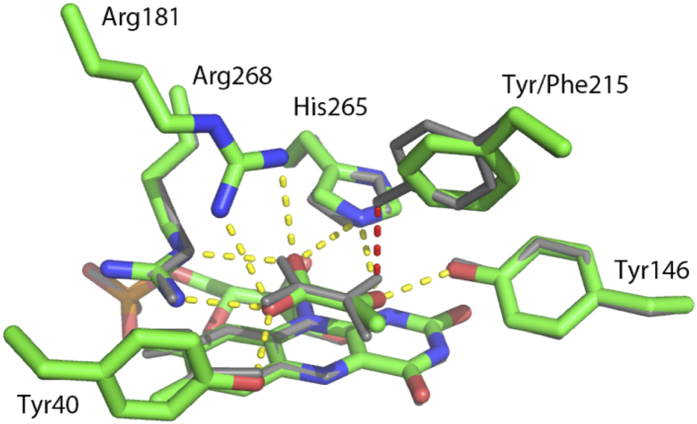
An overlay of the active sites from wild-type avLOX (grey) and the Y215F variant (colored by atom type) is shown. Hydrogen bonding in the wild-type structure is shown (yellow dashed line) including the hydrogen bond lost in the mutant (red dashed line). A subtle repositioning of the pyruvate and the phenylalanine side chain create a new hydrophobic contact in the mutant. This replaces a hydrogen bond with poor geometry between the tyrosine and pyruvate keto group.

The proposed reaction pathway for wild-type avLOX consists of two half-reactions, as shown in Fig. 1 1,11,12,13,37, in which FMN is first reduced by L-lactate (steps *k*_1_–*k*_6_) to become subsequently re-oxidized by O_2_ (step *k*_7_). The reductive half-reaction involves Michaelis complex formation, conversion to reduced enzyme-pyruvate complex and pyruvate release. Comparison of results between wild-type enzyme and the two variant lactate oxidases suggests that the reaction pathway was not altered by the substitution of Tyr^215^ with phenylalanine or histidine. Steady-state kinetic parameters are thus described with rate constants, and relevant rate constants were measured directly in stopped-flow experiments. Table 3 summarizes the results.

The stopped-flow parameter (*k*_obs_) associated with the main absorbance decrease at 455 nm during anaerobic enzyme reduction by L-lactate reflects the FMN reduction (step *k*_3_, *k*_4_). For each variant as shown in Fig. 4, the dependence of *k*_obs_ on the L-lactate concentration went through the origin. Therefore, this indicates^42^ that the “reverse” rate constant of FMN oxidation by pyruvate (*k*_4_) had a value not different from zero within the error limit. Expected consequence of *k*_4_ ≈ 0 s^−1^ is that the amplitude of the stopped-flow traces corresponds to a completely reduced enzyme, independent of the L-lactate concentration^35^,^42^. This was observed for both variants (Fig. 4) just like for the wild-type enzyme. The *k*_red_ found at saturating L-lactate concentration (Table 1) therefore equals the rate constant of FMN reduction *k*_3_. Effect on *k*_3_ of site-directed replacement of Tyr^215^ was relatively small in the Y215F variant (2.1-fold decrease) whereas it was large in the Y215H variant (33-fold decrease). In each enzyme, *k*_3_ was hardly affected (≤2-fold) by change in pH in the range 5.5–9.5.

**Figure 3:**
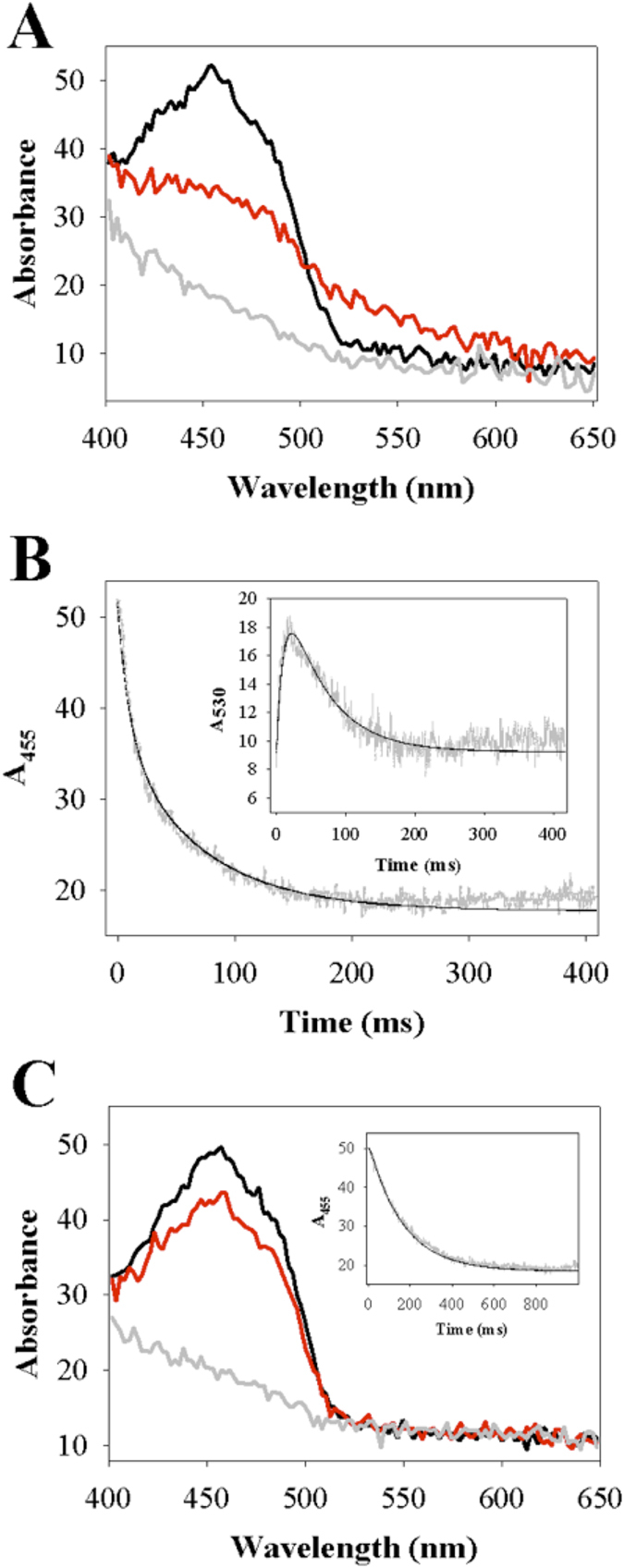
Stopped-flow kinetic analysis of anaerobic reduction of lactate oxidase variants Y215F (**A,B**) and Y215H (**C**) by L-lactate at 20 °C and pH 6.5 is shown. **A**) A superpositioning of wavelength scans of oxidized Y215F (4.3 μM) before (black line) and after 20 ms (red line) and 200 ms (grey line) of reaction with 10 mM L-lactate. The spectra show decrease in absorbance at 455 nm reflecting the FMN reduction but also intermittent increase in absorbance at around 530 nm reflecting accumulation of reduced enzyme-pyruvate complex. After reaction for 200 ms, absorbance at both 455 nm and 530 nm is lost, indicating that the enzyme was completely reduced and also the reduced enzyme-pyruvate complex was dissociated. **B**) A comparison of time traces of FMN reduction at 455 nm and reduced enzyme-pyruvate complex formation at 530 nm (inset). Experimental measurements are shown in grey and fits of the data are shown as black lines. The trace at 455 nm was fitted with a double exponential, the trace at 530 nm was described by a combined kinetic simulation and fitting using Fig. 1, which is described in the Discussion. **C**) A superpositioning of wavelength scans of oxidized Y215H (4.3 μM) before (black line) and after 20 ms (red line) and 500 ms (grey line) of reaction with 10 mM L-lactate. The spectra show decrease in absorbance at 455 nm to yield completely reduced enzyme, yet there is no intermittent increase in absorbance at around 530 nm. The inset shows a time trace of aborbance at 455 nm. Experimental data are shown in grey and a single exponential fit is shown as black line.

**Figure 4:**
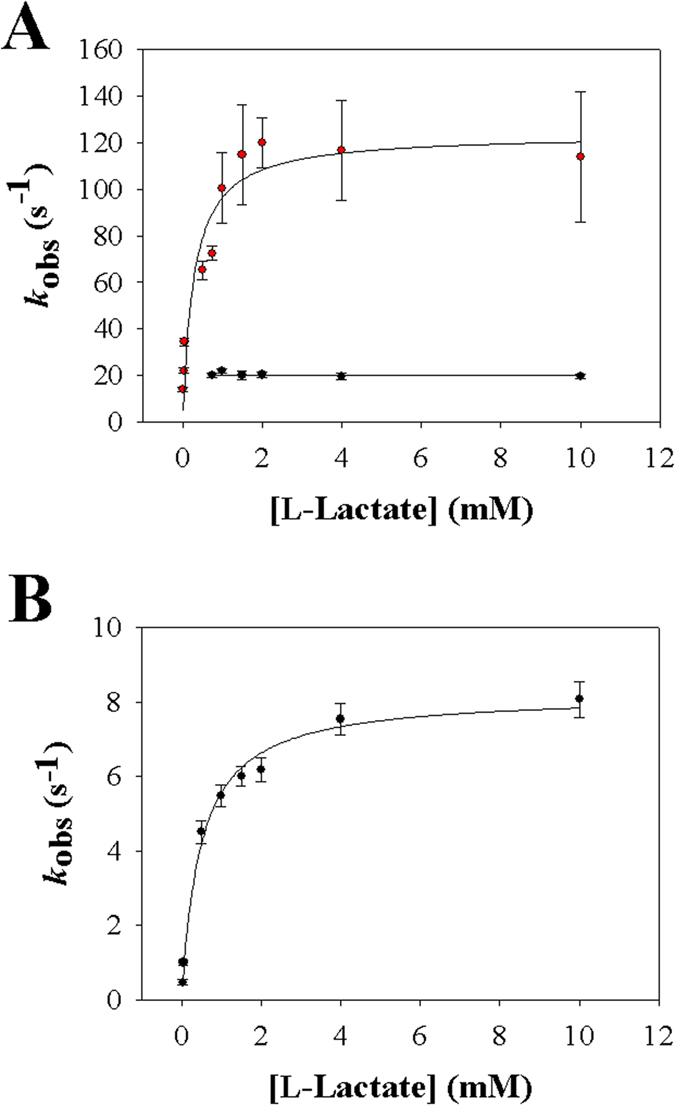
Analysis of stopped-flow rate constants of anaerobic reduction of lactate oxidase variants Y215F (**A**) and Y215H (**B**) by L-lactate at 20 °C and pH 6.5. **A**) The dependence of the rate constant of the fast FMN reduction phase (red symbols) on the L-lactate concentration. The black line is a hyperbolic fit of the data, yielding *k*_red_ and *K*_d_. The rate constant of the second (slow) phase of absorbance decay at 455 nm is shown in black symbols. It is independent of the L-lactate concentration and the line shows the constant (average) value. Note: the lowest L-lactate concentration at which the slow phase could be distinguished from the fast phase was around 0.75 mM. The line therefore stops at this concentration. **B**) The dependency of the rate constant of the single FMN reduction phase (black symbols) on the L-lactate concentration is shown. The black line is a hyperbolic fit of the data, yielding *k*_red_ and *K*_d_. Error bars show the S.D. from three or more independent determinations.

**Figure 5:**
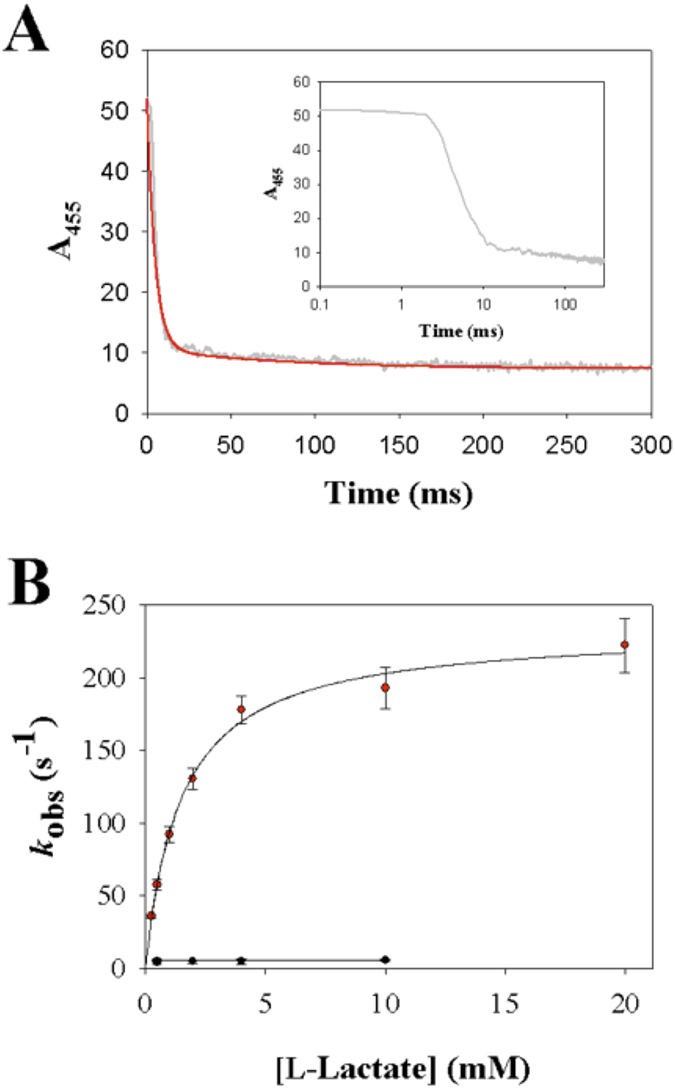
Stopped-flow kinetic analysis of anaerobic reduction of wild-type lactate oxidase by L-lactate at 20 °C and pH 9.5 is shown. In panel A is shown a time trace of absorbance at 455 nm on reaction of the enzyme (4.5 μM) with 10 mM L-lactate. The trace is biphasic where a fast phase in which about 90% of total absorbance change occurs (≤15 ms) is followed by a much slower phase of absorbance decrease. Measurements are shown in grey and double exponential fit of the data is shown in red. The inset shows a logarithmic plot of the data. In panel B is shown the analysis of the dependence of the stopped-flow rate constants on the L-lactate concentration. The rate constant of the second slow phase of absorbance decay was independent of the L-lactate concentration.

**Figure 6:**
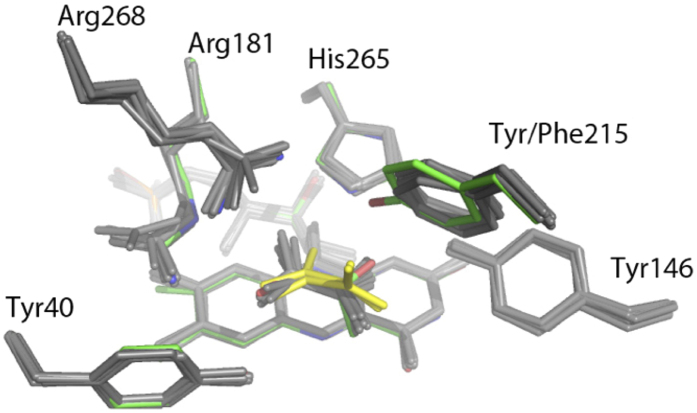
Superpositioning of the active sites from a wild-type monomer (colored by atom type) and the eight subunits from the Y215F mutant (grey) is shown. The pyruvates from the Y215F mutant subunits with an open lid (yellow) show that lid residues including Tyr^215^ constrain the binding of the pyruvate. The ring at the site of the mutation is slightly but consistently shifted from the conformation of the wild-type enzyme. This has the effect of substituting a van der Waals interaction between the phenyl group of Phe^215^ and the pyruvate methyl for a weak hydrogen bond between Tyr^215^ and the carbonyl oxygen in the wild-type enzyme.

The rate constant for pyruvate release (*k*_5_) was obtained directly from the stopped-flow traces when a second kinetic phase was present in the 455-nm time course (Y215F, Fig. 3; wild-type enzyme at pH 9.5, Fig. 5) or the reduced enzyme-pyruvate complex was immediately observable due to charge-transfer absorption at 530 nm (Y215F; Fig. 3). Of note, kinetic significance of the reduced enzyme-α-keto-acid complex is not a sufficient condition for detection of a charge-transfer band during enzyme reduction. Studies of avLOX^1^,^12^,^13^ and related α-hydroxyacid oxidases^43^,^44^ show that other factors (e.g. temperature) are also important. In wild-type avLOX, for example, the charge-transfer band is visible at 4 °C but not at 20 °C, despite the fact that *k*_5_ is the slowest step of the reaction at both temperatures^1^,^12^,^13^. Alternatively, in the case of wild-type enzyme at pH 5.5 and 6.5 or Y215H, k5, was obtained from the relationship (Equation 1) between the apparent *k*_cat_ (determined at air saturation; Table 1) and the reaction rate constants of Fig. 1. The *k*_7_ equals *k*_ox_ (Table 1) and [O_2_] was 250 μM.


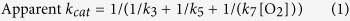


The *k*_5_ of Y215F (20.3 s^−1^) at pH 6.5 was lowered 7-fold compared to the corresponding *k*_5_ of the wild-type enzyme (141 s^−1^). The *k*_5_ of Y215H (26 s^−1^) was also decreased. Interestingly, however, replacement of Tyr^215^ by histidine affected the catalytic reduction of FMN (33-fold decrease) more strongly than it affected the pyruvate release (5.4-fold decrease). In the Y125F variant, by contrast, effect of the site-specific substitution on enzyme reduction by L-lactate was mainly on *k*_5_.

**Figure 7:**
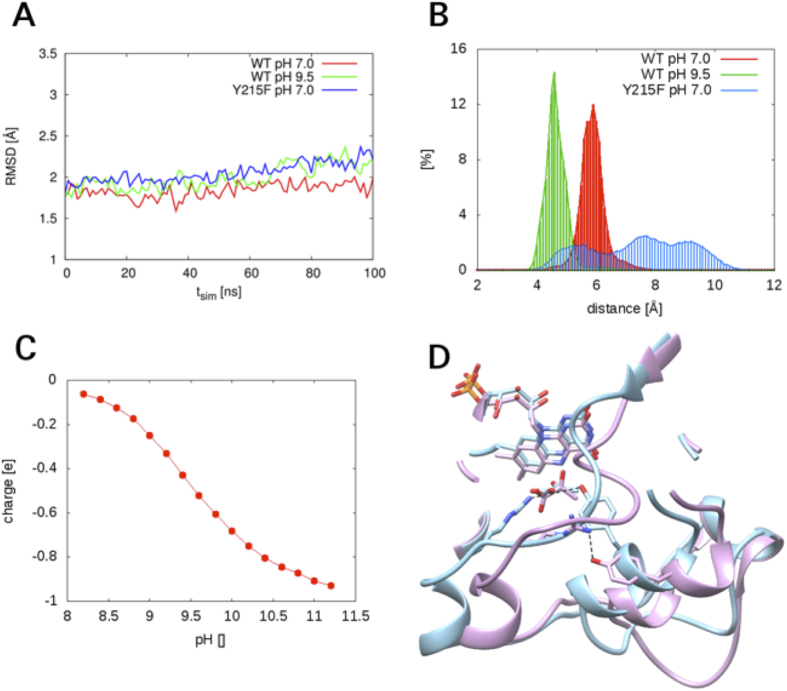
Results of MD simulations of wild-type and Y215F lactate oxidases are shown. (**A**) R.m.s.d. traces from the 100-ns simulations performed using the enzyme crystal structure (PDB code **2E77**) as reference. The experimental crystal structure was determined at pH 8.0. Note: a pre-equilibration time of 5 ns was used that is not shown in the graph. The r.m.s.d. traces therefore do not start at a value of zero. (**B**) The relative frequency distributions of distances between the guanidino Cζ of Arg^181^ and the phenyl Cζ of Tyr^215^/Phe^215^. (**C**) The calculated curve of pH-dependent ionization of Tyr^215^ in the (oxidized) enzyme-pyruvate complex of wild-type avLOX. (**D**) A superimpositioning of MD simulated structures of wild-type lactate oxidase at pH 7.0 (blue) and pH 9.5 (magenta). The structures shown are the final snapshots after 100 ns of MD simulation. The hydrogen bond between Arg^181^ and Tyr^215^ (set to be ionized at pH 9.5) is shown with a dashed line. The distance at pH 7.0 was 4.6 Å, that at pH 9.5 was only 2.9 Å.

Lowering the pH from the optimum value of 6.5 to 5.5 resulted in a slight (2.5-fold) decrease in *k*_5_ ( = 57 s^−1^) of the wild-type enzyme. Raising the pH to 9.5, however, caused a large (27-fold) drop in *k*_5_ to a value of 5.3 s^−1^. Considering the apparent *k*_cat_ ( = 4.1 s^−1^) of the enzyme under these conditions, the results imply pyruvate release to have become completely rate limiting at the pH of 9.5. The *k*_5_ of Y215F variant was essentially independent of pH in the range 5.5–9.5 (Table 1), clearly indicating that the high-pH dependence of *k*_5_ in wild-type enzyme had been eliminated completely as result of the substitution of Tyr^215^ by phenylalanine. Supported by evidence from MD simulations (Fig. 7) a plausible interpretation of these findings is that due to ionization of its side chain at high pH, Tyr^215^ develops a stronger interaction with Arg^181^ (Fig. 7B,D) and the interaction over the bound pyruvate is therefore tightened, thus explaining the large decrease in *k*_5_ under these conditions. Replacement of Tyr^215^ by phenylalanine was shown in the crystal structure and by MD simulation to result in complete disruption of this polar interaction. Evidence that *k*_5_ was nevertheless slowed down in the Y215F variant compared to wild-type enzyme at pH 6.5 could be explained by the development of new interactions between Phe^215^ and the bound pyruvate.

The rate constant of enzyme re-oxidation by O_2_ (*k*_7_ = *k*_ox_) was not strongly dependent on pH in the range 5.5–9.5 (≤3-fold change). Replacement of Tyr^215^ by histidine was by far more disruptive on *k*_7_ than was the replacement by phenylalanine. Reactivity of FMN in each catalytic step of L-lactate oxidation (*k*_3_, *k*_7_) is therefore affected by the active-site microenvironment due to residue 215.

Summarizing, Tyr^215^ has a dual role in facilitating the catalytic reaction of *A. viridans* lactate oxidase. It functions as a latch fastening a lid over the active site and is dynamically involved in a protein conformational change required for L-lactate binding and pyruvate release. Secondly, although direct interaction of Tyr^215^ with the bound L-lactate/pyruvate is shown to be not important for catalysis, the tyrosine appears to establish an active-site microenvironment in which FMN reduction and oxidation both proceed optimally, balancing the flux through each step in catalysis. Change to high pH or site-directed replacement of Tyr^215^ with phenylalanine disrupts this balance and creates a kinetic bottleneck at the pyruvate release step. The study thus reveals the subtle function of an auxiliary residue in the active site of lactate oxidase and it also suggests residues structurally homologous to Tyr^215^ as potential elements of evolutionary fine-tuning of reactivity in α-hydroxy acid oxidases.

## Methods

### Materials

Wild-type avLOX (grade 1) was a kind gift of Roche Diagnostics (Penzberg, Germany). The lyophilized protein was dissolved to about 10 mg/mL in 50 mM potassium phosphate buffer, pH 6.5. The avLOX migrated as a single protein band in SDS PAGE. It was therefore used without further purification. It was previously shown that the wild-type enzyme thus obtained behaved identically as the wild-type enzyme produced and purified by procedures described below for the variant lactate oxidases^12^. Glucose oxidase from *Aspergillus niger* (grade 1) was also from Roche. All other materials were described elsewhere^12^.

### Construction of Y215 variants, enzyme production and purification

Plasmid vector pLO-1 containing the avLOX gene under control of the tac promoter was used. A two-stage PCR protocol^45^ was applied in combination with suitable mutagenic oligonucleotide primers to introduce the desired mutation. Mutated avLOX genes were verified by sequencing.

Enzyme production was done in *Escherichia coli* BL21 (DE3) containing pLO-1 vector encoding wild-type or mutated avLOX. Cultivations of the strains, processing of *E. coli* cells, and protein purification (ammonium sulfate precipitation followed by hydrophobic interaction chromatography) were carried out by protocols exactly as described in our earlier paper^45^. Desalted protein was brought to about 20 mg/mL in 50 mM potassium phosphate buffer (pH 7.0) and stored at −70 °C.

### Assays

Roti-Quant assay referenced with BSA was routinely used to determine protein. The standard LOX activity assay was based on *in situ* peroxidase-coupled detection of the H_2_O_2_ released during L-lactate (50 mM) oxidation at pH 6.5. It was carried out at 37 °C as described elsewhere^45^. Initial rate studies of L-lactate oxidation were performed at 20 °C. The pH was varied in the range 5.5–9.5. Air-saturated potassium phosphate buffer (50 mM) was used. For determination of kinetic parameters, initial rates were recorded at 10–15 different L-lactate concentrations done in triplicate.

Absorbance spectra were recorded with a Varian Cary 50 Bio UV-Vis spectrophotometer using Hellma quartz cuvettes of 10 mm light path. An extinction coefficient of protein-bound FMN of 12500 M^−1^cm^−1^ was determined using a reported protocol^12^. Enzyme turnover numbers (*k*_cat_) are determined using the molar concentration of protein-bound FMN.

### Stopped-flow experiments

An Applied Photophysics model SX17MV stopped-flow analyzer equipped with a diode array absorbance detector was used to study the enzyme reductive and oxidative half-reactions. Alternatively, these experiments were performed by absorption measurements at a single wavelength (455 nm or 530 nm) recorded with a photomultiplier detector. Diode array data were recorded every 0.5 ms and the instrument dead time was below 1.5 ms under the conditions used. Data from diode array measurements (280–700 nm) were deconvoluted using Applied Photophysics Pro-K software. Anaerobic reaction conditions were established by flushing all solutions exhaustively with N_2_. Traces of oxygen were removed by adding 10 mM glucose and 200 nM glucose oxidase. All parts of the stopped-flow apparatus were rinsed with glucose/glucose oxidase solution. A 50 mM potassium phosphate buffer (pH 6.5) was used. The reductive half-reaction was also studied at pH 5.5 and at pH 9.5, also using 50 mM potassium phosphate. Due to low buffering capacity at these conditions, the pH was carefully set and shown to be stable during time of the reactions. Control reactions done in a universal buffer system (10 mM MES, 10 mM HEPES, 20 mM ethanolamine) gave consistent results.

The reductive half-reaction was followed by collecting spectra with the diode array detector or by measuring the decrease in absorption at 450 nm after mixing anaerobically the enzyme (5–20 μM final concentration) with varied concentrations of L-lactate at 20 °C. The oxidative half-reaction was followed also at 20 °C by mixing enzyme, which had been anaerobically reduced by a double molar equivalent of L-lactate, with varied concentrations of O_2_. Absorption at 455 nm was recorded with a photomultiplier detector. Single wavelength traces were fitted to the appropriate single or double exponential decay/rise function to obtain pseudo-first-order reaction rate constanst (*k*_obs_).

### Crystallization, structure determination, and refinement

Purified Y215F avLOX variant was crystallized using hanging drop vapor diffusion in air atmosphere at 20 °C. One μL of protein solution at 10 mg/mL in 50 mM potassium phosphate (pH 7.0) were mixed with 1 μL of reservoir solution of 30% polyethylene glycol, 50 mM Tris-HCl buffer (pH 8.0) and 50 mM pyruvate. Crystals were harvested in well solution supplemented with 20% (v/v) ethylene glycol. Diffraction data were collected at the Stanford Synchrotron Radiation Laboratory beamline 7-1. A spacegroup of P2_1_ was determined with unit cell parameters of a = 107.35 Å, b = 119.18 Å, c = 19.56 Å, β = 107.52°. Data were integrated using XDS^46^ then scaled using AIMLESS^47^. Molecular replacement using PHASER^48^ was done to position two LOX tetramers in the asymmetric unit using the avLOX Y191F variant structure (PDB code: **4YL2**)^37^ as a search object. Cycles of manual fitting and refinement using REFMAC5^49^ were done to reach a final R_cryst_ of 18.2% and an R_free_ of 24.6%. Iterations of refinement and manual refitting were used to identify and add waters, ethylene glycol and pyruvate molecules to the structure.

### Molecular dynamics simulation

MD simulations were performed on enzyme complexes with FMN and pyruvate in explicit solvent. PDB coordinates **2E77** (wild-type enzyme containing FMN and pyruvate) were used and missing residues 1–7 were modeled from enzyme bound to FMN alone (PDB: **2DU2**)^35^. The Amber99-SB-ILDN protein force field^50^ was used in combination with GAFF^51^ for FMN and pyruvate. Ligand partial charges were determined using the RESP algorithm combined with ab-initio HF/6-31G(d) calculations on the RED-server^52^. Partial charges of Tyr^215^ at pH 9.5 were calculated using the tri-peptide Ala-Tyr-Ala. Protonation states were evaluated using H++^53^. The TIP3P water model was used and sodium molecules were modeled with Aqvist parameters. Each system was solvated in approximately 18000 water molecules, plus the appropriate number of sodium ions to achieve zero net-charge. A cubic simulation box with a side length of approximately 8.5 nm was used. The Gromacs MD simulation engine and analysis programs version 4.6 were used^54^. A time step of 2 femtoseconds was used with a Verlet type integrator. Electrostatic long-range interactions were accounted for using the Particle Mesh Ewald method. Initial energy minimizations used equilibration runs for 5 ns at ambient conditions using Berendsen thermo and barostats. Production runs of up to 100 ns were performed using Nose-Hoover thermostat at 300 K and a Parrinello-Rahman barostat at 1 atm. Snapshots of the structures for subsequent analysis were saved at intervals of two picoseconds.

## Additional Information

**Accession codes:** The structure of the avLOX Y215F mutant has been deposited in the Protein Data Bank under accession number 5EBU.

**How to cite this article**: Stoisser, T. *et al.* Conformational flexibility related to enzyme activity: evidence for a dynamic active-site gatekeeper function of Tyr^215^ in *Aerococcus viridans* lactate oxidase. *Sci. Rep.* **6**, 27892; doi: 10.1038/srep27892 (2016).

## Supplementary Material

Supplementary Information

## Figures and Tables

**Table 1:** Stopped-flow kinetic parameters for L-lactate oxidation catalyzed wild-type avLOX and site-directed Y215 variants thereof.

Parameter	Enzyme								
	Wild-type avLOX			Y215F			Y215H		
	Reaction pH								
	5.5	6.5^c^	9.5	5.5	6.5	9.5	5.5	6.5	9.5
*k*_red_ (s^−1^)^a^^,^^b^	1.7 (±0.1) × 10^2^	2.7 (±0.2)^c^ × 10^2^	2.3 (±0.2) × 10^2^	62 (±5.5) [n.d.]^d^	1.2 (±0.1) × 10^2^ [1.5 (±0.1) × 10^2^]^d^	85 ± 7.3 [n.d.]^d^	7.2 ± 0.68	8.2 ± 0.75	8.7 ± 0.79
*K*_d_ (mM)^a^^,^^b^	3.0 ± 0.4	1.0 ± 0.1^c^	1.5 ± 0.1	n.d.	0.28 ± 0.03	n.d.	2.3 ± 0.18	0.55	0.20 ± 0.03
*k*_slow_ (s^−1^)^a^^,^^b^	n.a.	n.a.	5.3	12 ± 0.9 [11 ± 1.0]^d^	20 ± 1.7 [17 ± 1.5]^d^	20 ± 1.8 [18 ± 1.6]^d^	n.a.	n.a.	n.a.
*k*_ox_ (M^−1^s^−1^)^a^^,^^b^	1.1 (±0.1) × 10^6^	1.8 (±0.1)^c^ × 10^6^	6.0 (±0.2) × 10^5^	n.d.	7.0 (±0.4) × 10^5^	n.d.	n.d.	1.0 (±0.1) × 10^5^	n.d.

Results were obtained in 50 mM potassium phosphate buffer at 20 °C.

n.d., not determined; n.a., not applicable. A *k*_slow_ indicated as being “not applicable” means that a second slow phase of absorbance decrease at 455 nm was not observed in the stopped flow experiment.

^a^*k*_red_ is the FMN reduction rate constant which corresponds to the main (fast) phase of decrease in absorbance at 455 nm and is L-lactate concentration dependent. *K*_d_ is an apparent dissociation constant of L-lactate. *k*_slow_ is the rate constant for the second (slow) phase of absorbance decrease at 455 nm, independent of the L-lactate concentration. *k*_ox_ is the second-order rate constant of FMN re-oxidation by O_2_. It is explained in the Discussion that according to the proposed reaction pathway for wild-type and variant lactate oxidases (Figure 1), *k*_red_ corresponds to *k*_3_, *k*_slow_ corresponds to *k*_5_, and *k*_ox_ corresponds to *k*_7_.

^b^Parameters are from stopped-flow measurements at 455 nm. Traces were fitted with single or double exponential function and the resulting stopped-flow rate constants were analyzed for dependence of the varied substrate concentration (L-lactate, O_2_). Parameters are from the data in Figs 3, 4, 5 (*k*_red_, *k*_slow_) or Fig. S2 (*k*_ox_).

^c^The data for wild-type enzyme at pH 6.5 are from our previous paper^37^.

^d^Parameters shown in squared brackets are from stopped-flow measurements at 530 nm.

**Table 2:** Determination of the Y215F avLOX crystal structure: crystallographic data collection and refinement statistics are shown.

Data collection	
* *Unit cell dimensions	
* a, b, c* (Å)	107.35 119.18 119.56
* β* (°)	107.52
* *Resolution range (Å)	40.00–2.6 (2.74–2.6)
* *Completeness (%)	99.3 (96.8)
* *R_merge_	7.7 % (27.7 %)
* *Observations/unique reflections	329193/87748
* *Mean I/σ (I)	14.7 (4.8)
Refinement statistics	
* *R_cryst_ (%)	18.2
* *R_free_ (%)	24.6
* *R_free_ test set	2637
Deviations from ideal geometry	
* *Bond lengths (Å)	0.012
* *Bond angles (°)	1.5
Ramachandran Plot (%)	
* *Most favored	94.7
* *Additionally allowed	4.0
* *Outliers	1.3

Values in parentheses are for data in the high-resolution bin.

**Table 3:** Rate constants calculated from kinetic data in Table 1.

Rate constant	Enzyme								
	Wild-type avLOX			Y215F			Y215H		
	Reaction pH								
	5.5	6.5	9.5	5.5	6.5	9.5	5.5	6.5	9.5
*k*_3_ (s^−1^)^a^ FMN reduction	170^b^	270^b^	230^b^	62^b^	120^b^	85^b^	7.2^b^	8.2^b^	8.7^b^
*k*_5_ (s^−1^)^a^ Pyruvate release	57^c^ (65%)^f^	141^c^ (54%)^f^	5.3^d^ (95%)^f^	12^d^(n.d.)	20^d^ (78%)^f^	20^d^ (n.d.)	n.d. (n.d.)	26^c^(61%)^f^	n.d. (n.d.)
*k*_7_ [O_2_](s^−1^)^a^^,^^e^^,^^g^ FMN oxidation	275	450	162	n.d.	175	n.d	n.d.	25	n.d.

^a^Rate constant numbering is according to Fig. 1. The value of [O_2_] was 250 μM.

^b^*k*_3_ is the FMN reduction rate constant *k*_red_ (Table 1).

^c^*k*_5_ was determined from its relationship with the apparent *k*_cat_, derived from Equation 1: *k*_5_ = *k*_cat_app_/(1-*k*_cat_app_/*k*_3_-*k*_cat_app_/*k*_7_[O_2_]). *k*_cat_app_ is for reaction at [O_2_] = 250 μM.

^d^*k*_5_ is the rate constant for the second (slow) phase of absorbance decrease at 455 nm. See *k*_slow_ in Table 1.

^e^Note that *k*_7_ corresponds to *k*_ox_ (Table 1).

^f^Percent rate limitation in *k*_cat_app_ at 250 μM O_2_ due to *k*_5_.

^g^As shown in earlier studies^1^,^12^,^13^, the kinetic constants can be used to calculated the *k*_cat_ at saturating oxygen concentration and also the *K*_m_ for oxygen: *k*_cat_ = *k*_3_*k*_5_/(*k*_3_ + *k*_5_); *K*_m_ (O_2_) = *k*_cat_/*k*_7_.
