# Supplementary material for: Conformational flexibility related to enzyme activity: evidence for a dynamic active-site gatekeeper function of Tyr215 in Aerococcus viridans lactate oxidase
Source: Sci Rep. 2016 Jun 15;6:27892. doi: 10.1038/srep27892 (PMC4908395; doi:10.1038/srep27892)
Supplement: Supplementary Information [file srep27892-s1.doc]

**Conformational flexibility related to enzyme activity: evidence for a dynamic active-site gatekeeper function of Tyr^215^ in *Aerococcus viridans* lactate oxidase**

Thomas STOISSER^1,2^, Michael BRUNSTEINER^1^, David K. WILSON^3^, and Bernd NIDETZKY^1,2,*^

^1^ Research Center Pharmaceutical Engineering, Inffeldgasse 13, A-8010 Graz, Austria

^2^ Graz University of Technology, Institute of Biotechnology and Biochemical Engineering, NAWI Graz, Petersgasse 12, A-8010 Graz, Austria

^3^ Department of Molecular and Cellular Biology, University of California, Davis, CA 95616, USA

* Corresponding author (B.N.)

E-mail: bernd.nidetzky@tugraz.at; phone: +43 316 873 8400; fax: +43 316 873 8434


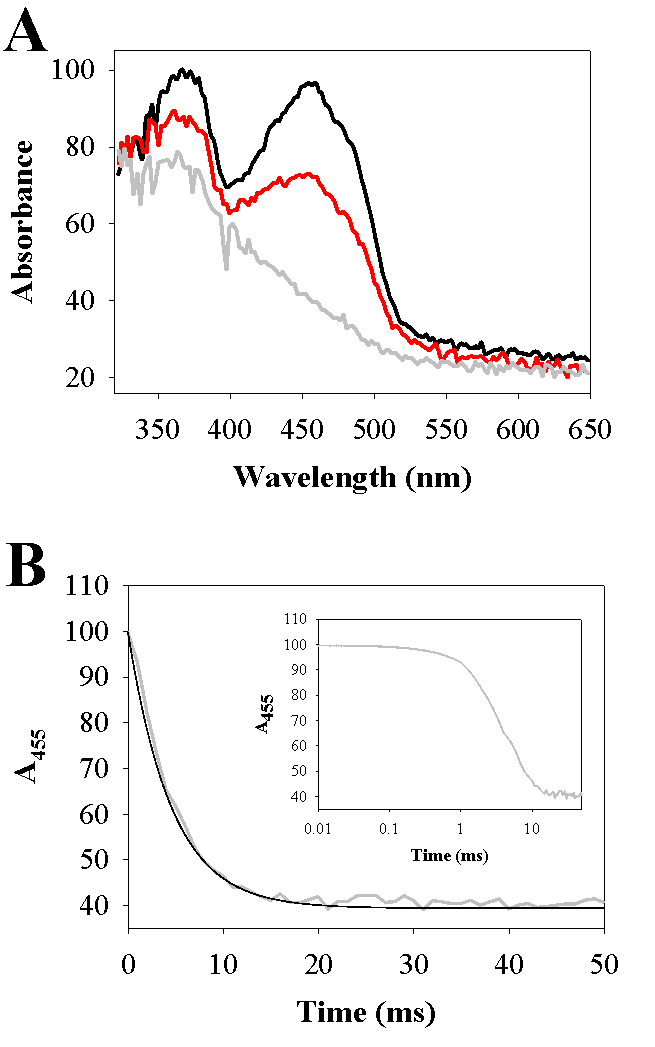


**Figure S1**. **Stopped-flow kinetic analysis of anaerobic reduction of wild-type lactate oxidase by L-lactate at 20 °C and pH 6.5 is shown.** A) A superpositioning of wavelength scans of oxidized enzyme (8 µM) before (black line) and after 20 ms (red line) and 200 ms (grey line) of reaction with 10 mM L-lactate. The spectra show decrease in absorbance at 455 nm reflecting the FMN reduction. After reaction for 200 ms, absorbance at 455 nm is lost, indicating that the enzyme was completely reduced. B) Time trace of FMN reduction at 455 nm (inset: log scale). Experimental measurements are shown in grey and fits of the data are shown as black lines.


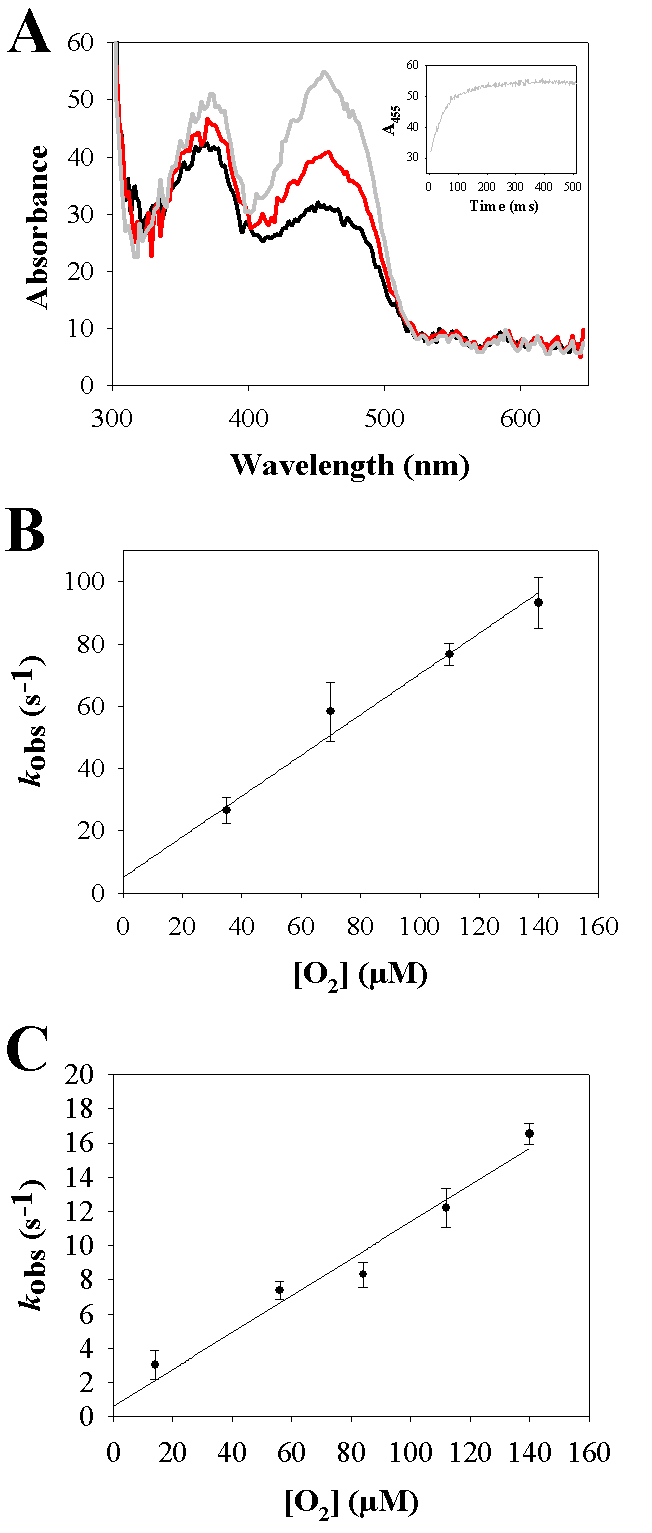


**Figure S2**. **Stopped-flow kinetic analysis of oxidation of reduced avLOX variants Y215F and Y215H by O_2_ at 20 °C and pH 6.5.** A) A superpositioning of wavelength spectra recorded from Y215H (5.3 µM) reduced anaerobically with two molar equivalents of L-lactate on oxidation with 140 µM O_2_ for 3 ms (black line), 35 ms (red line) and 300 ms (grey line). The enzyme is shown to become completely re-oxidized. The inset shows the time trace of absorbance at 455 nm (grey line) fitted with a single exponential function (black line). B, C) Dependencies of the stopped-flow rate constants of enzyme oxidation on the O_2_ concentration are shown. Symbols show the rate constants with S.D. from three or more independent experiments. The black lines are straight-line fits whose slopes correspond to the *k*_ox_ values summarized in Table 1 in main text.


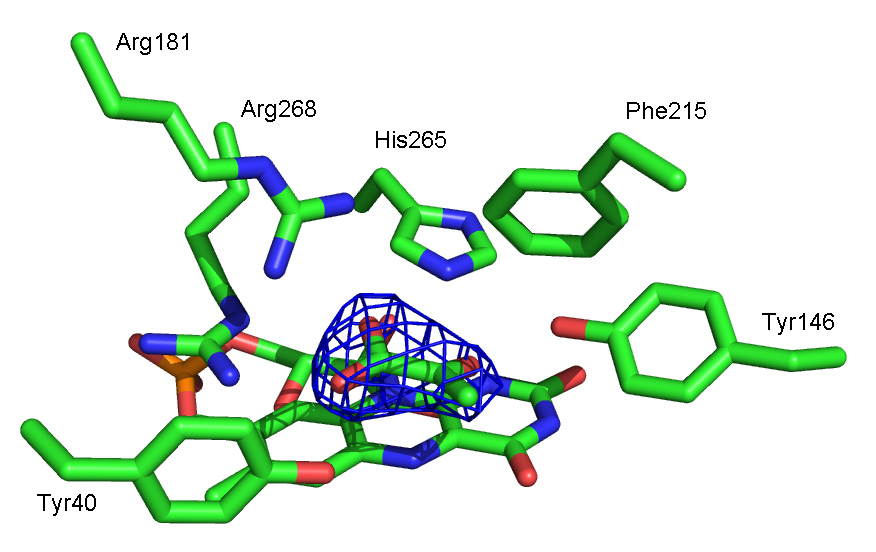


**Figure S3.** The active site of the Y215F variant in an orientation identical to Figure 1. The density shown is an |F_o_-F_c_| map calculated with phases derived from the final model with contributions from the pyruvate removed. The map is contoured at 3σ.
